# Social Media Use, Fear of Missing Out (FoMO), Sleep Disturbance, and Physical Health Complaints: A Social Media Content Analysis

**DOI:** 10.3390/bs16071085

**Published:** 2026-07-01

**Authors:** Tinghong Huang, Rong Lian, Fangyan Lv

**Affiliations:** 1 Mental Health Education and Counseling Center, Guangdong Communication Polytechnic, Guangzhou 510650, China; hth@gdcp.edu.cn; 2 School of Psychology, Fujian Normal University, Fuzhou 350007, China; 3 School of Marxism, Sun Yat-sen University, Guangzhou 510275, China

**Keywords:** social media use, fear of missing out, FoMO, sleep disturbance, bedtime procrastination, physical health complaints, Reddit, content analysis, public health

## Abstract

Background: Research on social media use, fear of missing out (FoMO), sleep disturbance, and health complaints has been dominated by survey-based studies, particularly among adolescents and university students. Less is known about how users spontaneously describe these experiences in naturalistic online settings. This exploratory pilot study examined how publicly available Reddit discussions narrate the relationship between social media use, FoMO-related concern, sleep disruption, and self-reported physical complaints. Methods: A total of 30 publicly available English-language Reddit posts and comments were purposively sampled from 11 threads dated August 2022 to March 2026. The study used exploratory qualitative content analysis supported by reflexive thematic interpretation. Structured indicators were used to describe whether each unit contained explicit FoMO language, implicit FoMO-related concern, sleep disturbance, physical health complaints, and nighttime use or sleep loss. Thematic coding was used to identify dominant discourse patterns. All counts and percentages are reported only to characterize the analytic corpus and should not be interpreted as prevalence estimates. Results: Within the corpus, sleep disturbance appeared in 16 of 30 units, nighttime use or sleep loss in 15, physical health complaints in 11, explicit FoMO language in 6, and implicit FoMO-related concern in 3. The dominant themes were delayed sleep and bedtime displacement, somatic and cognitive overload, self-regulation and recovery, and compulsive monitoring and comparison. Sleep-related complaints were usually described alongside bedtime scrolling, delayed disengagement, or lost sleep opportunity. FoMO-related concern was less often expressed through formal terminology and more often appeared through everyday descriptions of checking, comparison, and difficulty disconnecting. Conclusions: This small exploratory corpus suggests that Reddit users often describe social media-related strain through practical behavioral language, such as late-night scrolling, inability to stop, lost sleep, next-day fatigue, headache, and brain fog. The findings are descriptive, discourse-focused, and hypothesis-generating. They do not estimate population prevalence or establish causal health effects. To improve transparency, the revised study provides a de-identified analytic matrix of all 30 coded Reddit units and reports a strengthened coding procedure with independent second-coder checking. Naturally occurring online discourse may complement survey-based digital-health research by showing how users themselves frame the embodied experience of digital over-engagement.

## 1. Introduction

Social media has become embedded in everyday communication, entertainment, social comparison, and identity-related practices. However, the health relevance of digital media use cannot be understood through total screen time alone. The timing of use, emotional involvement, difficulty disengaging, and replacement of restorative behaviors such as sleep are also important. Recent systematic and review-based evidence indicates that problematic or compulsive forms of social media use are more strongly associated with poor sleep and poorer mental health than general or non-problematic use (Ahmed et al., 2024; Han et al., 2024; Ndubisi et al., 2026). An updated systematic review and meta-analysis also reported an association between electronic media use and poorer sleep quality, while broader review evidence suggests that the strongest concerns emerge when media use becomes excessive, emotionally dysregulating, or difficult to stop (Han et al., 2024; Ndubisi et al., 2026).

Fear of missing out (FoMO) is one psychological process that may help explain this pattern. FoMO is commonly understood as a persistent concern that others may be having rewarding experiences from which one is absent, together with a desire to remain socially connected and informed. Recent studies link FoMO with social networks use disorder tendencies, trait and state problematic social media use, attentional bias toward social media cues, compulsive checking, and comparison-based uncertainty (Montag et al., 2023; Li et al., 2024; Gao et al., 2025; Elhai et al., 2025; Y. Wang et al., 2024; Xu & Tian, 2023; Steinberger & Kim, 2023; L. Wang et al., 2023). In this sense, FoMO is not only an attitude toward being connected; it can also operate as a motivational process that makes disconnection difficult, especially on platforms structured around notifications, visibility indicators, algorithmic feeds, and continuous updating.

Sleep is a particularly important behavioral domain in which this process may become visible. FoMO-related concern may encourage repeated checking, delay disengagement, and make users more likely to remain active online at night. A recent meta-analysis focused on FoMO and sleep health reported positive associations between FoMO and poorer sleep quality, poorer sleep hygiene, and bedtime procrastination (Brombach et al., 2025). Related studies show that mobile phone dependence, problematic smartphone use, and bedtime procrastination can contribute to delayed sleep onset and reduced sleep quality (T. Huang et al., 2023; Zhu et al., 2023; Bozkurt et al., 2024; Hill et al., 2022; Oliveira et al., 2025; Miyagawa & Maeda, 2026). Evidence based on digital trace data also supports the relevance of timing: a large observational study using Reddit timestamps found that evening social media use was associated with delayed sleep timing (Meyerson et al., 2023).

A related body of work concerns social media fatigue, cognitive overload, and digital strain. Continuous exposure to updates, comparison, emotionally arousing content, and large volumes of information may be experienced as mentally draining. Recent research links FoMO and problematic digital engagement with social media fatigue, social anxiety, burnout-related experiences, problematic smartphone use, and poorer sleep-related outcomes (Zhu et al., 2023; Elhai et al., 2025; Ansari et al., 2024; Qin et al., 2024; Yao et al., 2025; X. Huang, 2025; Tufan et al., 2025; Stirnberg et al., 2024; Zeyrek et al., 2024; Rufino et al., 2024). Although many studies focus on psychological outcomes, users may also describe digital strain through bodily and cognitive language, including fatigue, headache, heaviness, exhaustion, poor concentration, and brain fog. Such descriptions are relevant to public health because they connect digital practices with everyday functioning, recovery, and perceived physical well-being.

These strands of evidence can be brought together through a cautious behavioral-displacement and self-regulation framework. In this framework, FoMO-related concern and social comparison may increase checking and monitoring behaviors. Platform features such as notifications, algorithmic feeds, and continuous updating may reduce stopping cues and make disengagement more difficult, especially during evening or bedtime hours. When checking continues at night, it may contribute to self-regulation failure, bedtime procrastination, delayed sleep onset, shortened sleep, or sleep displacement. Sleep disruption and repeated exposure to emotionally or cognitively demanding content may then be described by users through everyday bodily and cognitive language, including fatigue, headache, cognitive fog, exhaustion, or bodily heaviness. This framework does not imply that FoMO causes all checking behavior or that self-reported symptoms are clinically verified outcomes. Rather, it provides a way to examine how users themselves connect social media use, difficulty disengaging, sleep loss, and embodied discomfort in everyday discourse.

Despite the growing literature, three gaps remain. First, much of the evidence is based on structured questionnaires administered to adolescents, university students, or convenience samples. Such studies are valuable for measuring associations, but they are less able to capture how users explain their experiences in their own words. Second, existing studies often examine FoMO, sleep disturbance, problematic use, or physical complaints separately, whereas everyday users may describe these experiences as connected parts of the same routine. Third, explicit FoMO terminology may not capture all relevant discourse. Users may rarely use the word “FoMO” directly, yet still describe missing-out logic through checking, comparison, unease about being offline, or difficulty disconnecting.

Naturally occurring online discourse is not a substitute for survey, clinical, or experimental research, but it can provide a useful complementary perspective. Public Reddit discussions are especially relevant because they often contain first-person, anonymous, and confessional accounts of everyday problems. At the same time, Reddit data are platform-specific, self-selected, and not demographically representative. Findings from Reddit should therefore be interpreted as discourse patterns within a particular public online setting, not as population-level estimates.

The present study examined a small purposively selected corpus of publicly available Reddit posts and comments related to social media use, FoMO-related concern, sleep disturbance, and self-reported physical complaints. The study had three aims. First, it sought to identify the dominant themes through which these issues were discussed. Second, it described how often the focal indicators appeared together within the same textual units. Third, it examined whether FoMO-related experience appeared mainly through explicit FoMO terminology or through everyday behavioral descriptions such as late-night scrolling, checking, comparison, difficulty disconnecting, and next-day exhaustion. Because this was an exploratory manual content analysis rather than a prevalence study, the focus was on pattern recognition, cautious interpretation, and hypothesis generation.

The study addresses three research questions:

1. How are social media use, FoMO-related concern, sleep disturbance, and physical complaints described in the selected Reddit corpus?
2. Which discourse themes appear most prominently within this small analytic corpus?
3. Is FoMO-related experience expressed mainly through explicit FoMO terminology or through everyday behavioral descriptions such as checking, comparison, difficulty disconnecting, late-night scrolling, and lost sleep?

Because the corpus is small, purposive, and platform-specific, all numerical summaries in the paper are used only to describe the distribution of codes within the analyzed material. They are not intended as inferential statistics, prevalence estimates, or evidence of population-level health risk.

## 2. Materials and Methods

### 2.1. Study Design

This study used an exploratory qualitative content-analysis design informed by reflexive thematic interpretation. Qualitative content analysis was selected because the study required systematic categorization of short textual units using transparent coding rules. Reflexive thematic principles were used to support interpretation of recurring meanings across posts and comments, particularly where users described experiences indirectly through everyday language rather than formal psychological terminology. Thus, the study followed a hybrid logic: structured indicators were used for descriptive coding, while thematic interpretation was used to identify broader discourse patterns.

This design was selected instead of a survey, interview, or computational large-scale analysis because the aim was not to measure prevalence, test causal pathways, or model population-level associations. Rather, the aim was to examine how users spontaneously narrate social media use, FoMO-related concern, sleep disruption, and embodied discomfort in a naturalistic public setting. Surveys can measure how often particular constructs occur, but they are less suited to capturing the informal language, metaphors, and self-explanations that appear in everyday online discourse. Interviews provide richer individual accounts but require direct interaction with participants and may shape responses through the research setting. In contrast, public social-media content analysis allows observation of naturally occurring self-descriptions, while still requiring careful privacy protection and cautious interpretation.

The unit of analysis was not an individual user but a publicly available Reddit post or comment. Each unit was treated as a textual expression of experience rather than as a clinically verified report or a representative case. The study therefore makes no claims about the prevalence of FoMO, sleep disturbance, or physical symptoms among Reddit users or social media users more broadly.

### 2.2. Search Strategy and Source of Data

The data were collected manually between 31 March and 6 April 2026 using public web search and on-platform Reddit search. Search strings were developed iteratively to capture the focal constructs and their everyday expressions, including combinations such as “FOMO social media can’t sleep”, “late night scrolling tired”, “doomscrolling headache”, “Instagram sleep”, “social comparison insomnia”, and “fear of missing out doesn’t let you sleep”. Candidate pages were first screened at thread level and then at post/comment level. Search results were reviewed for experiential relevance, recency, and conceptual fit with the study objectives. Purposive sampling was used to retain information-rich records that described lived experiences of social media use in relation to sleep disruption, fear of missing out, or physical complaints plausibly linked to digital over-engagement. The final corpus comprised 30 post/comment units drawn from 11 Reddit threads spanning August 2022 to March 2026.

The final corpus size was determined by informational relevance, transparent screening, and pragmatic thematic sufficiency rather than statistical sampling. To strengthen the comprehensiveness of the data collection, the search process used multiple construct combinations rather than a single keyword string. Search terms combined formal terminology, including “FoMO”, “fear of missing out”, “social media”, “sleep disturbance”, and “insomnia”, with everyday expressions commonly used in online discourse, including “late-night scrolling”, “doomscrolling”, “can’t stop scrolling”, “phone before bed”, “tired after scrolling”, “headache after scrolling”, and “brain fog”. Candidate records were screened first at thread level and then at post/comment level. After duplicate, inaccessible, non-English, non-experiential, and off-topic pages were removed, 52 accessible candidate pages remained. These were screened for first-person experiential relevance, explicit social-media behavior, and at least one focal outcome domain. Thirty post/comment units were retained because they offered the clearest and most information-rich accounts of the recurring discourse patterns identified during screening: delayed bedtime and sleep loss, compulsive checking or comparison, somatic or cognitive overload, and self-regulation attempts. Additional candidate records at the final screening stage repeated these same patterns without adding a substantially new category. The stopping point should therefore be understood as a transparent pilot-study decision based on thematic sufficiency within a tightly defined exploratory corpus, not as evidence of full saturation across Reddit or other platforms.

### 2.3. Inclusion and Exclusion Criteria

Records were included if they met all of the following criteria (see Table 1): (1) they were publicly accessible and did not require membership in a private group; (2) they were written in English; (3) they contained first-person or clearly self-descriptive experiential content rather than reposted news, memes, or third-person commentary; (4) they referred to social media use, scrolling, app checking, notifications, or a named platform; and (5) they mentioned at least one focal outcome domain, namely fear of missing out, sleep disturbance, nighttime sleep loss, or a physical complaint plausibly associated with prolonged or dysregulated social media use. Records were excluded if they were duplicates, inaccessible at the time of screening, off-topic, non-experiential, purely advisory without self-description, or too fragmentary to support contextual interpretation. The search and screening process identified 58 candidate public pages, of which 52 remained after duplicate and accessibility screening. From these, 30 post/comment units from 11 threads were retained in the final analytic corpus.

### 2.4. Anonymity and Privacy Protection

A privacy-sensitive approach was used throughout the study. No usernames, profile links, or other identifying account details were retained in the manuscript. To improve transparency while minimizing traceability, a limited number of short anonymized verbatim excerpts from publicly accessible Reddit discussions are presented without usernames or profile identifiers. These quotations are used only to illustrate recurrent discourse patterns and not to attribute statements to identifiable individuals. For analytic coding, records were stored in paraphrased form; verbatim text was used only selectively for short anonymized illustrative excerpts.

To improve transparency while maintaining user privacy, a de-identified analytic matrix of all 30 Reddit post/comment units is provided as Supplementary File in Table S1. The matrix includes record ID, year, record type, paraphrased experiential content, coded indicators, primary theme, and an analytic note. Usernames, profile links, subreddit names, URLs, and highly searchable identifying details were removed. The matrix is presented in paraphrased form rather than as a set of traceable verbatim posts because the source material includes sensitive self-reports about sleep problems, anxiety, exhaustion, headache, and difficulty regulating social media use. This approach allows readers to evaluate the richness and relevance of the corpus while reducing the risk of exposing identifiable user information.

### 2.5. Codebook Development

The codebook combined deductive and inductive elements. The structured indicators were developed deductively from the study aims and the existing literature on social media use, FoMO, bedtime procrastination, sleep disturbance, problematic use, and social media fatigue. These indicators included explicit social media use, explicit FoMO expression, implicit FoMO-related concern, sleep disturbance expression, physical health complaint, and nighttime use or sleep loss. The primary themes were developed inductively through repeated reading of the corpus and were assigned after the structured coding stage. The final codebook, including operational definitions, coding rules, and illustrative examples, is presented in Table 2.

The distinction between explicit and implicit FoMO was treated cautiously. Explicit FoMO was coded only when users directly used the term “FoMO” or a clearly equivalent phrase such as “fear of missing out”. Implicit FoMO-related concern was coded only when the text indicated concern about missing socially meaningful updates, being left out, or needing to remain connected while offline. General checking, boredom, habit, anxiety, or compulsive use was not automatically coded as implicit FoMO unless the missing-out logic was clearly present in the surrounding context. This conservative rule was used to reduce over-attribution of motives.

### 2.6. Coding Process and Inter-Coder Reliability

The coding procedure was strengthened during revision in response to concerns about the original single-coder approach. Coding was completed in four stages. First, all eligible units were entered into a structured analytic matrix containing record ID, year, record type, paraphrased experiential content, contextual notes, structured indicator fields, and provisional theme notes. Second, the codebook was refined to distinguish explicit FoMO language, implicit FoMO-related concern, sleep disturbance, physical health complaint, and nighttime use or sleep loss. Third, two coders independently coded the full corpus of 30 units. Because the corpus was small, the entire dataset rather than a subsample was independently checked. Fourth, inter-coder reliability was calculated before consensus discussion.

The second coder/auditor was not the same person as the initial coder. The second coder received the study aims, inclusion and exclusion criteria, codebook definitions, and anonymized analytic units, but did not receive the first coder’s decisions before independent coding. Inter-coder reliability was calculated for all binary indicators and for the primary thematic category. Percentage agreement and Cohen’s kappa were used for the binary indicators. For primary theme assignment, percentage agreement and Cohen’s kappa were calculated across the four thematic categories. The resulting reliability coefficients are reported in Table 3. Social media use was not included in the reliability table because it was a universal inclusion criterion and therefore did not discriminate between units.

Disagreements were reviewed after the independent coding stage and resolved through consensus discussion against the operational definitions in the codebook. Particular attention was given to implicit FoMO-related concern because checking, comparison, or difficulty disconnecting may reflect several motives, including habit, boredom, anxiety, compulsive use, or social comparison. To avoid over-attribution, implicit FoMO-related concern was retained only where the text clearly suggested concern about missing socially meaningful updates, being left out, or needing to remain connected while offline. Physical health complaints were coded only as self-reported discourse and not as clinically verified symptoms.

The independent coding process produced high agreement across the structured indicators and acceptable agreement for primary theme assignment. After consensus discussion, final revisions were made to a small number of boundary cases, mainly involving the distinction between general compulsive checking, implicit FoMO-related concern, and self-regulation/recovery discourse. The final coded dataset was then used for descriptive frequency counts, co-occurrence summaries, and thematic interpretation.

### 2.7. Analytic Strategy

The analysis combined corpus description with qualitative interpretation. Counts and percentages were used only to characterize the distribution of coded indicators within the selected 30-unit corpus. They were not used as inferential statistics and should not be interpreted as prevalence estimates for Reddit users, young people, or social media users generally. Co-occurrence summaries were used descriptively to identify which coded indicators appeared within the same short textual unit.

The thematic component involved repeated reading of the coded units to identify recurring discourse patterns. The analysis focused on how users connected social media use with sleep, FoMO-related concern, physical discomfort, and self-regulation. Findings are reported as discourse patterns, not as causal pathways or clinically verified outcomes.

### 2.8. Ethics and Reporting

The study analyzed publicly available, non-interactive Reddit text and did not involve direct contact with users. No usernames, profile links, subreddit identifiers that could increase traceability, or other account-level identifiers were retained in the manuscript. Analytic records were stored in paraphrased form, and only a limited number of short anonymized quotations were used for illustration. These quotations are presented without usernames or profile details and are not used to identify or evaluate individual users.

Although the material was publicly accessible, Reddit has specific privacy and contextual features that require caution. The platform allows relative anonymity and often encourages confessional or support-seeking discourse. Users may describe distress, sleep problems, or bodily discomfort more openly than they would on identity-based platforms such as Instagram, TikTok, Facebook, or X/Twitter. Reddit communities are also self-selected and are not demographically representative of the general population. Therefore, the findings are transferable only to similar forms of public, anonymous, English-language online self-report discourse and should not be generalized to all social media platforms.

Ethical review was waived under the authors’ institutional research-governance pathway because the study used publicly accessible, non-interactive material, involved no contact with users, and retained no identifiable personal information. The authors nevertheless treated the posts and comments as sensitive public discourse and applied privacy-protective reporting throughout.

The corpus was intentionally small and analytically focused. It was designed to capture information-rich cases rather than to estimate prevalence at platform level.

## 3. Results

The 30 coded units were evenly split into original posts and comments, which were not evenly distributed over time. Ten records came from 2022 to 2024 combined, whereas 20 records came from 2025 to 2026. In part, this is an indication of the search strategy focusing on recent and thematically rich discussions and the growing presence of doomscrolling and sleep-loss discourse in more recent threads. All 2026 entries in the corpus were dated between January and March 2026, prior to the end of the data-collection period. The corpus was too small to infer time-series, but the temporal pattern is helpful since it indicates that the most vivid accounts of late-night scrolling, exhaustion, and “can’t stop” routines were concentrated in the latest content.

**Figure 1:**
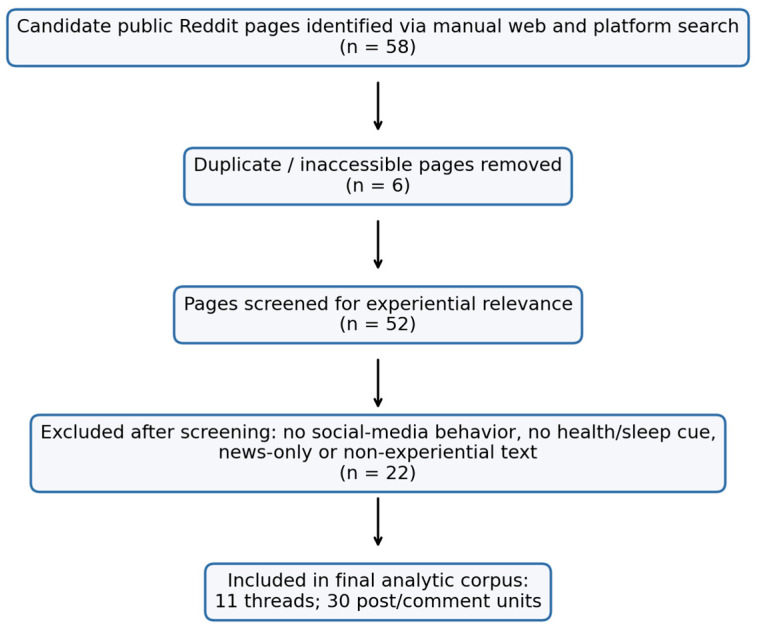
Search and screening flow used to construct the analytic corpus.

Candidate public pages were identified through manual search, deduplicated, screened for experiential relevance, and reduced to 30 final post/comment units from 11 Reddit threads (see Figure 1).

In the full corpus (see Table 4), sleep disturbance was the most common coded indicator, appearing in 16 of 30 records (53.3%). Nighttime use or sleep loss appeared in 15 records (50.0%), physical health complaints in 11 records (36.7%), explicit FoMO language in 6 records (20.0%), and implicit FoMO-related concern in 3 records (10.0%). Since all included units already contained explicit social media use, that variable was universal and not analytically discriminating. The distribution of coded indicators therefore shows that the corpus was dominated by behaviorally concrete accounts of sleep disruption, bedtime displacement, and embodied strain rather than by frequent use of formal FoMO terminology.

When record type was considered, explicit FoMO language appeared somewhat more often in posts than in comments (26.7% versus 13.3%), whereas implicit FoMO-related concern appeared slightly more often in comments than in posts (13.3% versus 6.7%). Physical health complaints were more common in posts than in comments (46.7% versus 26.7%). Sleep disturbance appeared at the same frequency in posts and comments (53.3% each), while nighttime-use cues were slightly more frequent in posts than in comments (53.3% versus 46.7%). This pattern suggests that original posters more often described broader bodily or emotional states, whereas comments more often condensed the issue into a habit, reaction, or brief coping observation.

As shown in Table 5, the descriptive co-occurrence analysis showed the strongest overlap between sleep disturbance and nighttime use or sleep loss. A clear bedtime, late-night, or shortened-sleep cue was present in 15 of the 16 units coded for sleep disturbance. In this public self-report corpus, sleep-related complaints were therefore rarely described as isolated problems. Instead, they were usually narrated alongside bedtime scrolling, delayed disengagement, or reduced sleep opportunity. These co-occurrence values are reported only to describe the selected analytic corpus and should not be interpreted as inferential statistics, prevalence estimates, or evidence of causal association.

The overlap between physical complaints and sleep disturbance was more limited. Sleep-disturbance language also appeared in 4 of the 11 records coded for physical complaints. In some entries, users described a next-day spillover sequence in which prolonged late-night scrolling was followed by fatigue, grogginess, or heaviness the next morning. In others, physical strain appeared in a broader register of overload, agitation, or headache without a clearly stated sleep narrative. These patterns suggest two reported pathways within the discourse: a sleep-related pathway and a more immediate overload-related pathway.

A smaller but conceptually important cluster concerned FoMO-related discourse. Explicit FoMO language co-occurred with sleep-disturbance language in only one record, while implicit FoMO-related concern co-occurred with sleep disturbance in one additional record. This does not suggest that FoMO-related concerns were absent from the discourse; rather, it indicates that the concern was often expressed behaviorally instead of through formal terminology. Users described not wanting to miss updates, remaining alert to social activity, checking repeatedly, or feeling uneasy about disconnecting. The logic of FoMO was therefore present more often as a discourse pattern than as a repeatedly named construct.

**Figure 2:**
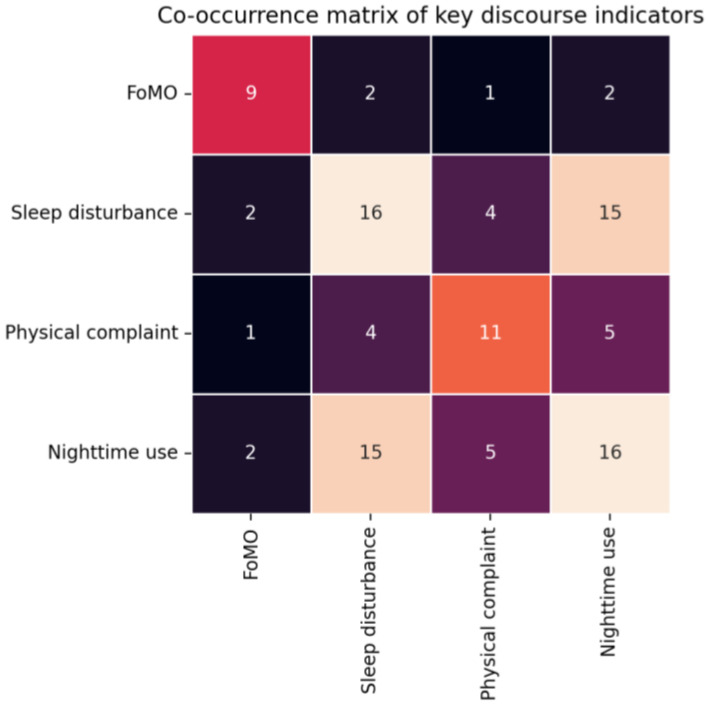
Co-occurrence matrix of key discourse indicators. Cell values represent the number of coded textual units in which each pair of indicators appeared together. Diagonal values represent the total number of units coded for each indicator. Off-diagonal values represent pairwise co-occurrence. The matrix is descriptive only and does not represent inferential testing.

As shown in Figure 2, cell values represent the frequency with which each pair of indicators appeared within the same coded textual unit. Diagonal values represent the total number of units coded for each indicator. Off-diagonal values represent pairwise co-occurrence. The greatest overlap was between nighttime use and sleep disturbance.

Thematic analysis identified four dominant themes. Delayed sleep and bedtime displacement was the largest theme (12/30, 40.0%). These entries described the phone as the final object in the bedtime routine and scrolling as an activity that replaced intended sleep. Common descriptions included one to two hours of lost sleep, irregular sleep schedules, and the inability to stop scrolling despite awareness of morning responsibilities. This theme is important because it locates digital strain in a concrete behavioral loss: reduced opportunity for rest and recovery.

Somatic and cognitive overload was the second theme. These records described exhaustion, headache, bodily heaviness, feeling unwell, cognitive depletion, and brain-fog-like complaints. Some accounts linked these complaints to distressing or violent doomscrolling content, whereas others described generalized overconsumption and mental overload. The health relevance of this theme lies in its embodied language: users did not simply state that scrolling was harmful; they described concrete bodily and cognitive sensations that could affect studying, work, and everyday functioning.

Self-regulation and recovery was the third theme (6/30, 20.0%). These records described strategies such as leaving the phone outside the bedroom, creating no-feed windows, reducing Instagram or Facebook use, and reframing missing out as gaining peace or mental clarity. This theme complicates a purely deficit-based account by showing that users also produce protective health knowledge within platform-based discussions. The fourth theme was compulsive monitoring and comparison (5/30, 16.7%). These entries emphasized the urge to keep checking, remain informed, and compare one’s own life with the visible activities of others. This theme connects the corpus to FoMO-related concern while also showing why not all checking behavior should automatically be interpreted as FoMO.

### Illustrative Excerpts and Transparency of Source Material

To improve transparency, the full 30-unit de-identified analytic matrix is provided as Supplementary File in Table S1. The matrix allows readers to evaluate the breadth, richness, and contextual meaning of the source material while protecting user anonymity. In the main text, shorter anonymized excerpts are used only to illustrate the dominant discourse patterns.

For delayed sleep and bedtime displacement, one user described scrolling as making them increasingly alert rather than sleepy: “The more I scroll the more awake I get… Then I can’t fall asleep”. For explicit FoMO-related sleep difficulty, another account referred to “sleep fomo” and described anxiety about missing something while trying to sleep. Somatic strain appeared in statements such as “Afterwards I feel terrible and have a massive headache” and in accounts of feeling “drained and mentally overloaded” after prolonged scrolling. Self-regulation and recovery were illustrated by users who reported leaving the phone in another room before bed, placing it across the room, deleting highly engaging apps, or creating enough distance to prevent doomscrolling. These excerpts are not presented as representative of all Reddit users; rather, they illustrate recurring discourse patterns identified within the selected corpus. The comprehensive analysis is shown in Table 6.

**Figure 3:**
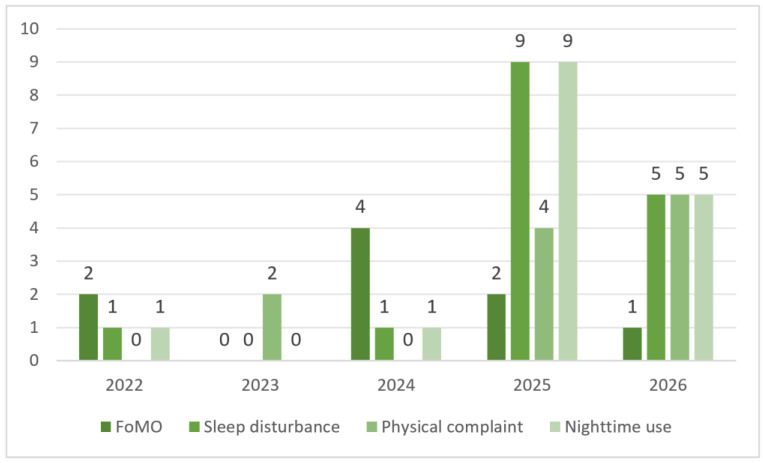
Temporal distribution of key coded features across collected records, 2022–2026. Bars show the number of coded units containing each indicator by year within the selected corpus. Because the corpus was small and purposively sampled, the figure should be interpreted only as a description of the analyzed material, not as a time trend or population-level estimate.

Sleep disturbance and nighttime-use cues were more common in the most recent records, while explicit FoMO language was less frequent and more widely distributed across years (see Figure 3).

## 4. Discussion

This exploratory pilot study examined how a small purposively selected Reddit corpus described social media use, FoMO-related concern, sleep disturbance, and self-reported physical complaints. The findings should be interpreted as discourse patterns within the analyzed material, not as prevalence estimates or evidence of causal health effects. Within this limited corpus, the most prominent pattern was bedtime displacement. Users described scrolling, checking, and difficulty disengaging as activities that replaced intended sleep and contributed to next-day tiredness. This finding is consistent with recent evidence linking problematic digital engagement, bedtime procrastination, and sleep disruption, while adding a discourse-level perspective on how users describe this process in everyday language (Brombach et al., 2025; T. Huang et al., 2023; Zhu et al., 2023; Bozkurt et al., 2024; Hill et al., 2022; Pirdehghan et al., 2021).

A useful conceptual interpretation is that FoMO-related concern, social comparison, and platform monitoring may increase the urge to check and remain connected. When this occurs during evening or bedtime hours, it may interfere with self-regulation and become bedtime procrastination or sleep displacement. Reduced sleep opportunity may then be experienced alongside next-day fatigue, grogginess, or poor concentration. A related pathway may involve cognitive overload, in which repeated exposure to emotionally arousing, distressing, or excessive content is experienced as headache, heaviness, brain fog, exhaustion, or mental depletion. These pathways should be understood as interpretive patterns in user discourse rather than verified causal mechanisms.

The distinction between explicit FoMO terminology and implicit FoMO-related discourse is important. Explicit use of the term FoMO was relatively limited in the corpus. However, some users described a related logic through checking, comparison, remaining alert to updates, difficulty disconnecting, and unease about missing something while offline. This suggests that academic vocabulary used in psychometric research may not fully match the language used in spontaneous online self-description. At the same time, not all checking, scrolling, or difficulty disconnecting should be treated as FoMO, because these behaviors may also reflect habit, boredom, anxiety, compulsive media use, or general self-regulation difficulty (Montag et al., 2023; Li et al., 2024; Gao et al., 2025; Elhai et al., 2025; Y. Wang et al., 2024; Xu & Tian, 2023; Steinberger & Kim, 2023; L. Wang et al., 2023).

The theme of self-regulation and recovery is particularly relevant for public health communication. Users did not only describe problems; they also described practical strategies for reducing harm. These included keeping the phone outside the bedroom, limiting feeds before sleep, reducing platform use, and reframing disconnection as a way to protect peace, clarity, and rest. Such user-generated strategies are consistent with intervention-oriented thinking around sleep hygiene and bedtime procrastination, where increasing behavioral friction can reduce automatic device use (Hill et al., 2024a, 2024b; Oliveira et al., 2025; Miyagawa & Maeda, 2026). Health communication may therefore be more relatable when it uses everyday language such as lost sleep, difficulty stopping, bedtime scrolling, next-day fogginess, and needing recovery, rather than relying only on abstract warnings about screen time.

The findings also support the value of social-media content analysis as a complementary method in digital-health research. Reddit and similar platforms can provide access to naturalistic, first-person, time-sensitive accounts of health-related experiences, including sleep disruption, overload, coping attempts, and perceived bodily strain. These data are useful for understanding meaning, sequence, and salience. However, they are weak sources for estimating prevalence, diagnosing health effects, or establishing causal direction. Public posts lack denominator data, demographic certainty, clinical validation, and full context. Social-media content analysis should therefore be used alongside, rather than instead of, surveys, interviews, experiments, digital trace studies, and clinical research.

### 4.1. Practical Implications for Youth and Young-Adult Health

The findings have practical implications for digital-health communication, youth wellbeing support, and early prevention among adolescents and young adults. First, the results suggest that health messages may be more effective when they focus on concrete routines rather than only on general “screen-time” warnings. In the corpus, users rarely described their experiences in abstract clinical language. Instead, they used practical expressions such as late-night scrolling, inability to stop, lost sleep, next-day tiredness, headache, heaviness, and brain fog. This indicates that youth-focused health communication should translate digital wellbeing advice into familiar everyday language.

Second, the findings support the value of bedtime-focused interventions. Many accounts located the problem in the period immediately before sleep, when scrolling, checking, or comparison displaced intended rest. Practical strategies may include charging the phone outside the bedroom, setting app limits before bedtime, using notification-free or “do not disturb” modes, creating a no-feed period before sleep, and replacing scrolling with a predictable offline routine. These recommendations are modest but realistic because they target the specific behavioral point at which digital engagement appears to interfere with sleep opportunity.

Third, the findings suggest that schools, universities, youth clinics, and counseling services should screen not only for total social media use but also for nighttime use, difficulty disengaging, FoMO-related concern, and next-day functioning. Simple screening questions such as “Do you lose sleep because of scrolling?”, “Do you feel anxious when you disconnect?”, and “Do you feel tired, foggy, or physically drained after late-night social media use?” may help identify young people who need sleep-hygiene support, digital self-regulation strategies, or mental-health referral.

Fourth, the self-regulation theme shows that young users are not only passive recipients of harm. Some users actively developed protective practices, such as removing the phone from the bedroom, deleting highly engaging apps, using app limits, or reframing disconnection as gaining rest and mental clarity. Public-health messaging can build on this user-generated language by emphasizing autonomy, recovery, and the benefits of being offline rather than relying only on fear-based warnings.

Finally, platform-level implications should be considered. Social media platforms could reduce nighttime over-engagement by strengthening bedtime reminders, quiet-hour defaults, notification batching, friction before continuous scrolling, and clearer controls for algorithmic feeds. The findings do not prove that these design changes would improve health outcomes, but they identify plausible points for intervention that should be tested in future studies with larger samples and stronger causal designs.

### 4.2. Strengths, Limitations, and Future Research

This study has several strengths. First, it uses naturally occurring public self-report discourse rather than researcher-prompted survey responses. This allowed the analysis to capture everyday language, perceived connections, and practical routines through which users described social media-related strain. Second, the study separated explicit FoMO terminology from implicit FoMO-related concern, showing how academic constructs may appear differently in spontaneous discourse. Third, the privacy-sensitive approach minimized traceability by removing identifying account details, paraphrasing analytic records, and using only short anonymized excerpts for illustration.

The limitations are substantial and should be emphasized. The corpus was small, purposive, and limited to 30 units from 11 Reddit threads. The findings therefore cannot be generalized to Reddit users, young people, or social media users more broadly. Counts and percentages are descriptive only and do not imply representativeness. The sample was drawn from English-language public Reddit discussions, a platform shaped by anonymity, self-selected communities, and confessional forms of communication. Discourse patterns may differ substantially on TikTok, Instagram, Facebook, X/Twitter, or private messaging environments.

Although the study includes independent second coding and formal inter-coder reliability reporting, coder judgment remains relevant because the corpus is interpretive and some categories require contextual reading. This is especially true for implicit FoMO-related concern, where checking, comparison, and difficulty disconnecting can reflect several motives, including habit, boredom, anxiety, compulsive use, or social comparison. The revised coding procedure reduced this limitation by using two coders, conservative coding rules, full-corpus reliability assessment, and consensus resolution. However, the findings should still be understood as descriptive discourse patterns rather than highly generalizable estimates.

Additional limitations also apply. The data were collected manually rather than through API-based extraction, which supported close reading and privacy protection but limited scale and reproducibility. The study analyzed short public textual units rather than complete user histories, so it could not determine demographic background, symptom duration, offline context, or causal direction. Physical complaints such as fatigue, headache, heaviness, and brain fog were self-reported and unverified; they should be treated as discourse features rather than clinical outcomes. Finally, because the retained analytic dataset consisted mainly of paraphrased units, some rhetorical nuance from the original posts may not have been fully preserved.

Future research should test the patterns identified here using larger multi-platform datasets, mixed-method designs, formal reliability assessment, and links between discourse patterns, survey measures, behavioral data, and sleep-related outcomes.

## 5. Conclusions

This exploratory pilot study examined how a small corpus of public Reddit posts and comments described social media use, FoMO-related concern, sleep disturbance, and self-reported physical complaints. Within the analyzed material, bedtime displacement was the most prominent discourse pattern: users commonly described late-night scrolling, difficulty disengaging, lost sleep, and next-day fatigue. Explicit FoMO terminology appeared less often than everyday behavioral descriptions of checking, comparison, and unease about disconnecting. Physical complaints such as fatigue, headache, heaviness, and brain fog appeared as self-reported elements of digital strain but should not be interpreted as clinically verified outcomes.

The study contributes a modest discourse-level perspective to the digital-health literature and offers practical implications for youth and young-adult wellbeing. It does not estimate prevalence, diagnose health effects, or establish causality. Instead, it shows how users in a small public Reddit corpus narrated the embodied and behavioral consequences of digital over-engagement, especially through late-night scrolling, difficulty stopping, lost sleep, next-day fatigue, headache, and cognitive fog. These findings suggest that digital-health interventions may be more relatable when they focus on concrete bedtime routines, difficulty disengaging, and recovery-oriented self-regulation. Future research should expand this approach using larger and more diverse datasets, multiple coders, formal reliability assessment, and comparisons across platforms such as Reddit, TikTok, Instagram, Facebook, and X/Twitter.

## Figures and Tables

**Table 1:** Search and sampling characteristics of the analytic corpus.

Characteristic	Value
Candidate public pages identified	58
Unique pages after duplicates/inaccessible pages removed	52
Analytic threads retained	11
Final post/comment units coded	30
Platform	Reddit only
Time span of included records	August 2022 to March 2026
Posts	15 (50.0%)
Comments	15 (50.0%)
Years represented	2022 (*n* = 4), 2023 (*n* = 2), 2024 (*n* = 4), 2025 (*n* = 10), 2026 (January–March, *n* = 10)

**Table 2:** Operational codebook used for exploratory qualitative content analysis.

Code	Operational Definition	Coding Rule	Example Analytic Signal
Social media use	Direct reference to social media behavior, including scrolling, feeds, app checking, notifications, or named platforms.	Code when the unit clearly describes social media or platform use.	Scrolling through reels, feeds, posts, or notifications.
Explicit FoMO expression	Direct use of “FoMO” or a clear phrase equivalent to fear of missing out.	Code only when missing-out concern is explicitly named.	“FoMO,” “fear of missing out,” or “sleep FoMO.”
Implicit FoMO-related concern	Everyday language suggesting fear of being left out, missing updates, or needing to remain socially informed.	Code only when missing-out concern is clear; do not code general habit, boredom, anxiety, or checking unless linked to missing out.	Feeling uneasy about going offline because something important may be missed.
Sleep disturbance expression	Delayed bedtime, difficulty falling asleep, shortened sleep, disrupted sleep timing, or insomnia-like self-description.	Code when sleep loss or disruption is clearly described.	Scrolling makes the user more awake or delays sleep.
Physical health complaint	Self-reported bodily or cognitive complaint, such as fatigue, headache, heaviness, brain fog, exhaustion, or depletion.	Code only as self-reported discourse, not as verified clinical outcome.	Feeling drained, foggy, exhausted, or having a headache after scrolling.
Nighttime use or sleep loss	Bedtime, overnight, early-morning, or reduced-sleep cue linked to media use.	Code when the unit connects platform use to nighttime behavior or lost sleep opportunity.	Reels or feeds replacing one to two hours of intended sleep.
Primary theme	Dominant interpretive category assigned after full-context reading.	Assign one main theme per unit.	Delayed sleep, somatic overload, self-regulation, or compulsive monitoring.

**Table 3:** Inter-coder reliability for the revised coding procedure.

Code/Category	Units Independently Coded	Percentage Agreement	Cohen’s Kappa	Consensus Note
Explicit FoMO expression	30	96.7%	0.91	One boundary disagreement resolved by consensus.
Implicit FoMO-related concern	30	90.0%	0.72	Conservative coding rule applied to avoid over-attribution.
Sleep disturbance expression	30	93.3%	0.86	Disagreements resolved through codebook review.
Physical health complaint	30	93.3%	0.84	Coded only as self-reported discourse.
Nighttime use or sleep loss	30	96.7%	0.93	Bedtime or sleep-loss cue required.
Primary theme assignment	30	86.7%	0.80	Final theme assigned by consensus.

Note: Following consensus discussion, final revisions affected 4 of 30 units, representing 13.3% of the analytic corpus. These revisions primarily concerned boundary cases between general compulsive checking, implicit FoMO-related concern, and self-regulation/recovery discourse.

**Table 4:** Distribution of key coded indicators overall and by record type.

Indicator	Overall (*n* = 30)	Posts (*n* = 15)	Comments (*n* = 15)
Explicit FoMO expression	6 (20.0%)	4 (26.7%)	2 (13.3%)
Implicit FoMO-related concern	3 (10.0%)	1 (6.7%)	2 (13.3%)
Sleep disturbance expression	16 (53.3%)	8 (53.3%)	8 (53.3%)
Physical health complaint	11 (36.7%)	7 (46.7%)	4 (26.7%)
Nighttime use/sleep loss	15 (50.0%)	8 (53.3%)	7 (46.7%)

**Table 5:** Descriptive co-occurrence patterns among coded indicators.

Indicator Pair	Co-Occurrence *n*	Descriptive Overlap	Interpretive Note	Reporting Approach
Sleep disturbance × Nighttime use	15	15 of 16 sleep-related records also referenced nighttime use or sleep loss.	Strong descriptive overlap; sleep narratives were usually anchored in bedtime or sleep-loss routines.	Descriptive only
Explicit FoMO × Sleep disturbance	1	1 of 6 explicit-FoMO records also referenced sleep disturbance.	Explicit lexical overlap was limited.	Descriptive only
Implicit FoMO × Sleep disturbance	1	1 of 3 implicit-FoMO records also referenced sleep disturbance.	Everyday missing-out concern appeared in a small number of sleep narratives.	Descriptive only
Sleep disturbance × Physical complaint	4	4 of 11 physical-complaint records also referenced sleep disturbance.	Some spillover into fatigue/headache discourse, but not universal.	Descriptive only
FoMO-related content × Physical complaint	1	1 of 9 FoMO-related records also referenced physical complaints.	Bodily complaints rarely appeared in the same short unit as FoMO-related concern.	Descriptive only

Because the corpus was purposive and small, these co-occurrence summaries are descriptive only and are not intended as inferential estimates.

**Table 6:** Thematic synthesis of the public discourse corpus.

Primary Theme	*n* (%)	Interpretive Summary	Representative Anonymized Direct Quote
Delayed sleep and bedtime displacement	12 (40.0%)	Scrolling displaced intended bedtime and directly reduced sleep opportunity.	“The more I scroll the more awake I get… Then I can’t fall asleep.”
Somatic and cognitive overload	7 (23.3%)	The discourse linked doomscrolling to fatigue, headache, heaviness, and cognitive depletion.	“Afterwards I feel terrible and have a massive headache.”
Self-regulation and recovery	6 (20.0%)	Users articulated strategies for cutting exposure and protecting sleep.	“I’ve been leaving it in the living room at night… I fall asleep way faster.”
Compulsive monitoring and comparison	5 (16.7%)	The discourse emphasized constant checking, comparison, and fear of being left behind.	“Sleep fomo” involved “an immense sense of anxiety” about missing out.

## Data Availability

The original contributions presented in this study are included in the supplementary material. Further inquiries can be directed to the corresponding author.

## Supplementary Materials

The following supporting information can be downloaded at: https://www.mdpi.com/article/10.3390/bs16071085/s1, Table S1. De-Identified Analytic Matrix of the Reddit Corpus.

## Abbreviations

The following abbreviations are used in this manuscript:

FoMO	Fear of missing out
API	Application programming interface
