# Supplementary material for: Social Media Use, Fear of Missing Out (FoMO), Sleep Disturbance, and Physical Health Complaints: A Social Media Content Analysis
Source: Behav Sci (Basel). 2026 Jul 1;16(7):1085. doi: 10.3390/bs16071085 (PMC13405858; doi:10.3390/bs16071085)
Supplement: Supplementary file 1 [file behavsci-16-01085-s001.zip › behavsci-4279312-supplementary.pdf]

## Supplementary File

This supplementary file presents the de-identified analytic matrix used for the exploratory qualitative content analysis. Usernames, profile links, subreddit names, URLs, and highly searchable identifying details have been removed to reduce traceability. Each unit is presented in paraphrased form rather than as a fully traceable verbatim extract. The matrix is intended to support transparency while protecting the privacy of users who posted publicly about sensitive experiences involving social media use, sleep disturbance, FoMO-related concern, and self-reported physical or cognitive complaints.

**Table S1. De-Identified Analytic Matrix Of The Reddit Corpus**

| Record ID | Year | Record type | De-identified/paraphrased experiential content | Explicit FoMO | Implicit FoMO-related concern | Sleep disturbance | Physical health complaint | Nighttime use/sleep loss | Primary theme | Analytic note |
|---|---|---|---|---|---|---|---|---|---|---|
| R01 | 2022 | Post | The user described being awake around 4:30 a.m. despite needing to attend class in a few hours. They linked the delay to phone scrolling after midnight, exhaustion, health strain, and difficulty disengaging. | No | No | Yes | Yes | Yes | Delayed sleep and bedtime displacement | Strong example of scrolling replacing intended sleep. |
| R02 | 2022 | Comment | The user reported not bringing the phone into the bedroom because it created too much temptation, and suggested using an alarm clock instead. | No | No | No | No | No | Self-regulation and recovery | Practical friction strategy to prevent bedtime scrolling. |
| R03 | 2022 | Comment | The user described keeping the phone across the room because having it nearby would lead to late-night scrolling. | No | No | Yes | No | Yes | Delayed sleep and bedtime displacement | Shows environmental control of bedtime phone use. |
| R04 | 2022 | Comment | The user recommended having no electronics in the bedroom and treating the bedroom as a rest-only space. | No | No | No | No | No | Self-regulation and recovery | Sleep-hygiene style advice based on removing devices. |
| R05 | 2023 | Post | The user described using a phone or computer before bed, staying awake 45 minutes to several hours longer than intended, feeling | No | No | Yes | Yes | Yes | Delayed sleep and bedtime displacement | Clear bedtime procrastination pattern. |

| | | | | | | | | | |
|---|---|---|---|---|---|---|---|---|---|
| | | | overstimulated, and not getting enough sleep. | | | | | | | |
| R06 | 2023 | Comment | The user reported deleting TikTok and Instagram to stop bedtime doomscrolling and replacing the routine with reading before bed. | No | No | No | No | No | Self-regulation and recovery | App deletion and reading used as replacement behavior. |
| R07 | 2024 | Post | The user described “sleep FoMO,” deleting social media apps to prevent scrolling, anxiety about missing something, racing thoughts, and sleeping around morning hours. | Yes | No | Yes | No | Yes | Delayed sleep and bedtime displacement | Explicit FoMO linked with severe sleep delay. |
| R08 | 2024 | Comment | The user interpreted sleep-FoMO as anxiety when others fall asleep before them and identified with that anxiety. | Yes | No | Yes | No | No | Compulsive monitoring and comparison | Explicit FoMO-related anxiety around sleep. |
| R09 | 2024 | Comment | The user stated that they could not stop scrolling generally, but avoided social media close to bedtime and used less stimulating text-based content to fall asleep faster. | No | No | Yes | No | Yes | Self-regulation and recovery | Differentiates social media from lower-stimulation media. |
| R10 | 2024 | Comment | The user described being able to remain in bed for very long periods while doomscrolling and needing timers or physical separation from the phone. | Yes | No | Yes | No | Yes | Delayed sleep and bedtime displacement | “Not missing anything” logic and time-loss pattern. |
| R11 | 2025 | Post | The user asked how to stop using the phone before sleep because they repeatedly scrolled for much longer than intended. | No | No | Yes | No | Yes | Delayed sleep and bedtime displacement | Short but direct bedtime scrolling problem. |
| R12 | 2025 | Comment | The user suggested that inability to put the phone down before bed may be due to FoMO, anxiety, depression, procrastination, habit, or avoidance. | Yes | No | No | No | No | Compulsive monitoring and comparison | Explicitly names FoMO as one possible mechanism. |

| | | | | | | | | | | |
|---|---|---|---|---|---|---|---|---|---|---|
| R13 | 2025 | Comment | The user described leaving the phone outside the bedroom by 10 p.m., reading or watching something low-stimulation, and falling asleep faster. | No | No | Yes | No | Yes | Self-regulation and recovery | Phone removal associated with improved sleep onset. |
| R14 | 2025 | Comment | The user suggested that scrolling may reflect a need to feel connected through social media. | No | Yes | No | No | No | Compulsive monitoring and comparison | Implicit connectedness/missing-out concern. |
| R15 | 2025 | Comment | The user reported removing interesting apps from the phone, keeping only limited social media access, realizing there was no reason to lose sleep over social media, and not feeling that anything important was missed. | No | Yes | Yes | No | Yes | Self-regulation and recovery | Recovery from anticipated missing-out logic. |
| R16 | 2025 | Post | The user described endless video watching and doomscrolling as preventing real activities such as music, walking, family interaction, and hobbies, and associated the routine with headache. | No | No | No | Yes | No | Somatic and cognitive overload | Somatic complaint linked with endless scrolling. |
| R17 | 2025 | Post | The user described Instagram as overstimulating after a short period of scrolling and reported developing a headache. | No | No | No | Yes | No | Somatic and cognitive overload | Headache linked with platform overstimulation. |
| R18 | 2025 | Comment | The user reported getting a headache when seeing a partner doomscroll through social-media content. | No | No | No | Yes | No | Somatic and cognitive overload | Observational but still self-reported bodily response. |
| R19 | 2025 | Post | The user described deleting social media for a period of time and experiencing improved sleep, including the absence of very late-night doomscrolling. | No | No | Yes | No | Yes | Delayed sleep and bedtime displacement | Sleep improvement after reducing social media. |

| | | | | | | | | | | |
|---|---|---|---|---|---|---|---|---|---|---|
| R20 | 2025 | Post | The user stated that evening wind-down often turned into scrolling through reels until very late at night, sometimes around 3 a.m., despite intending to sleep earlier. | No | No | Yes | No | Yes | Delayed sleep and bedtime displace ment | Reels-based bedtime displacement . |
| R21 | 2026 | Comme nt | The user described staying up at night doomscrolling and then feeling too tired to complete planned morning exercise. | No | No | Yes | No | Yes | Delayed sleep and bedtime displace ment | Next-day functioning affected by late-night scrolling. |
| R22 | 2026 | Post | The user described feeling drained and mentally overloaded after scrolling and YouTube use throughout the day, with the experience almost causing a headache. | No | No | No | Yes | No | Somatic and cognitive overload | Strong cognitive overload and headache pattern. |
| R23 | 2026 | Post | The user described trying phone-distance strategies and app limits but still returning to scrolling half-asleep, feeling zombie-like, and experiencing headache. | No | No | Yes | Yes | Yes | Delayed sleep and bedtime displace ment | Combines nighttime use, failed self-control, and headache. |
| R24 | 2026 | Post | After sharply reducing social media use, the user described recurrent headaches and wondered whether these symptoms were linked to withdrawal from scrolling. | No | No | No | Yes | No | Somatic and cognitive overload | Physical complaint framed around reduced use. |
| R25 | 2026 | Post | The user distinguished ordinary digital communication from infinite scrolling, describing scrolling as making them deeply unwell and mentally drained, while also feeling unable to delete Instagram because many friends communicate there. | No | Yes | No | Yes | No | Compulsi ve monitorin g and comparis on | Implicit social connection concern plus mental drain. |
| R26 | 2026 | Comme nt | The user reported that doomscrolling made the | No | No | No | Yes | No | Somatic and | Brain-fog/cognitive |

| | | | | | | | | | | |
|---|---|---|---|---|---|---|---|---|---|---|
| | | | brain feel fried and damaged attention for the rest of the day. | | | | | | cognitive overload | depletion discourse. |
| R27 | 2026 | Comment | The user described doomscrolling and Reddit reading as draining, tiring, annoying, and anxiety-provoking, especially when the content was triggering. | No | No | No | Yes | No | Somatic and cognitive overload | Emotional and bodily depletion after scrolling. |
| R28 | 2026 | Post | The user described quitting social media but struggling at night because they felt bored, kept thinking about reinstalling apps, and found sleep difficult. | No | No | Yes | No | Yes | Delayed sleep and bedtime displacement | Nighttime withdrawal from habitual scrolling. |
| R29 | 2026 | Post | The user discussed FoMO in relation to digital minimalism and described a long-standing feeling of not wanting to sleep because the day felt incomplete. | Yes | No | Yes | No | Yes | Delayed sleep and bedtime displacement | Explicit FoMO linked with resistance to sleep. |
| R30 | 2026 | Post | The user asked whether FoMO is stronger among generations shaped by the internet and social media, and contrasted constant online awareness with being unconcerned about missing out. | Yes | No | No | No | No | Compulsive monitoring and comparison | Explicit FoMO and social-media comparison discourse. |

**Summary of coded indicators:**

| Indicator | Frequency |
|---|---|
| Explicit FoMO expression | 6/30 |
| Implicit FoMO-related concern | 3/30 |
| Sleep disturbance expression | 16/30 |
| Physical health complaint | 11/30 |
| Nighttime use or sleep loss | 15/30 |

**Summary of primary themes:**

| Primary theme | Frequency |
|---|---|

| | |
|---|---|
| Delayed sleep and bedtime displacement | 12/30 |
| Somatic and cognitive overload | 7/30 |
| Self-regulation and recovery | 6/30 |
| Compulsive monitoring and comparison | 5/30 |
